# Characterization and intervention impacts on litter across public land types in Northern Idaho

**DOI:** 10.1007/s10661-026-15342-w

**Published:** 2026-04-28

**Authors:** Mary Engels, Mandira Panta

**Affiliations:** Natural Resources & Society, University of Idaho, Moscow, ID USA

**Keywords:** Litter, Recreation areas, Camp hosts, Plastic waste, Camping, Public lands

## Abstract

Litter in recreational areas presents a growing management challenge as public land visitation increases, yet it remains critically understudied compared to urban and marine environments. This study provides the first systematic characterization of litter composition and accumulation patterns in US inland recreational campgrounds, examining four Idaho State Parks and one national forest site during the 2021 camping season. We collected and analyzed 4834 litter items (13.5 kg) from 42 campsites and 3-day use areas, revealing a distinctive waste profile that differs markedly from other environments: plastic comprised 78.6% of litter, substantially exceeding proportions found in roadways and traditional waste streams. Critically, we demonstrate that campgrounds support urban-level population densities (median 4252 people/km^2^) yet maintain remarkably low litter accumulation rates (2.13–2.18 kg/km^2^/day) compared to urban settings. This study is the first to quantify the pivotal role of volunteer camp hosts in litter management, finding they removed an estimated 111,150 pieces (> 277 kg) annually from Idaho State Parks alone, representing over $171,600 in cost savings versus paid labor. In contrast, day use areas without dedicated volunteer cleaning exhibited significantly higher litter levels, establishing volunteer presence as a critical variable in recreational litter management. Our findings challenge assumptions about the inevitability of litter accumulation in high-density recreational settings and provide the first evidence-based framework for volunteer-driven litter management strategies in resource-constrained public land agencies.

**Supplementary Information:**

The online version contains supplementary material available at 10.1007/s10661-026-15342-w.

## Introduction

Public lands in the USA, at both state and federal levels, attract millions of visitors annually for recreational activities. However, mismanaged waste, including illegal dumping and littering, presents a persistent management challenge for officials working to maintain the quality of services that these lands provide. This problem has become increasingly urgent as recreational demand continues to grow and visitor numbers have surged since the COVID-19 pandemic (Idaho Department of Parks and Recreation, 2018; Chow, 2020; Blevins, 2025).

Litter creates aesthetic, environmental, and financial costs for both communities and public lands (Bator et al., 2011; Ojedokun & Balogun, 2011). The increased visitation to beaches, hiking trails, campgrounds, and harbors has led to greater litter accumulation along trails and overflow at garbage collection sites (Chow, 2020). Since public access to these areas is economically crucial for states, addressing litter management is essential for maintaining sustainable recreational opportunities.

Understanding the scope of litter requires clear definitions. Law et al. (2020) define litter as solid waste that enters the environment through intentional or unintentional disposal, occurring even where waste management infrastructure exists, and representing approximately 2% of global waste generation. This includes both active littering, where individuals purposefully leave waste behind, and passive littering, where waste is left despite efforts to prevent it (Liu & Shibley, 2004). Litter ranges from small items like cigarette butts and food wrappers to larger discarded appliances, furniture, or vehicles—essentially anything found in an “unacceptable location, regardless of its origin” (Schultz et al., 2013, p. 2).

The magnitude of the litter problem is substantial. Keep America Beautiful (KAB) estimates approximately 50 billion pieces of litter exist in US roads and waterways—nearly 152 pieces per US resident (KAB, 2021). While their latest report shows significant reductions in litter over time (61% decrease between 1969–2009 and an additional 54% decline since 2009), a slight uptick occurred from 2019 to 2020 due to pandemic-related increases in recreational use and associated littering behavior. A statewide litter survey by the Washington State Department of Ecology (2023) confirms decreases along interstate roadways between 1999 and 2022, but documented litter increases in State and County parks during that same period.

Waste and litter in recreational areas, such as parks, is a common, but understudied, management issue. Pierno (2017) estimates that US national parks alone generate over 100 million pounds of managed waste annually through operations and visitor activities which exclude mismanaged waste like litter. Limited research has attempted to quantify litter specifically in recreational settings, but available studies reveal concerning patterns. For example, state parks in New Jersey had the highest weekly accumulation rates of the five specialized environments (roadways, beaches, waterways, state parks, and landfills) surveyed. (Cutter et al., 1991). Recent reports demonstrate persistent litter issues in protected areas despite managed waste systems, such as 150 pounds of trash from illegal camping in Arches National Park (Will, 2021) and over 14,000 pieces collected by volunteers across 44 federal sites (5 Gyres, 2022).

As campgrounds and recreational areas continue to develop as spaces where visitors can enjoy natural landscapes, heavy use brings increased litter concerns. When littering dominates these areas, it reduces recreational experiences for visitors and can create conflicts among users (Brown et al., 2010). Understanding the quantity and types of litter left behind by recreationists can provide crucial insights for park management to develop effective anti-littering strategies while promoting positive waste management behaviors.

This study examines litter found in four state parks and one national forest recreation area across Northern Idaho to address three key questions: (1) What kinds of litter are left behind in recreational areas? (2) What management actions help reduce litter in recreational areas? (3) How much litter escapes into the environment despite existing management actions? By answering these questions, this research aims to provide evidence-based recommendations for improving litter management in recreational settings.

## Methods

### Study sites and characteristics

**Fig. 1:**
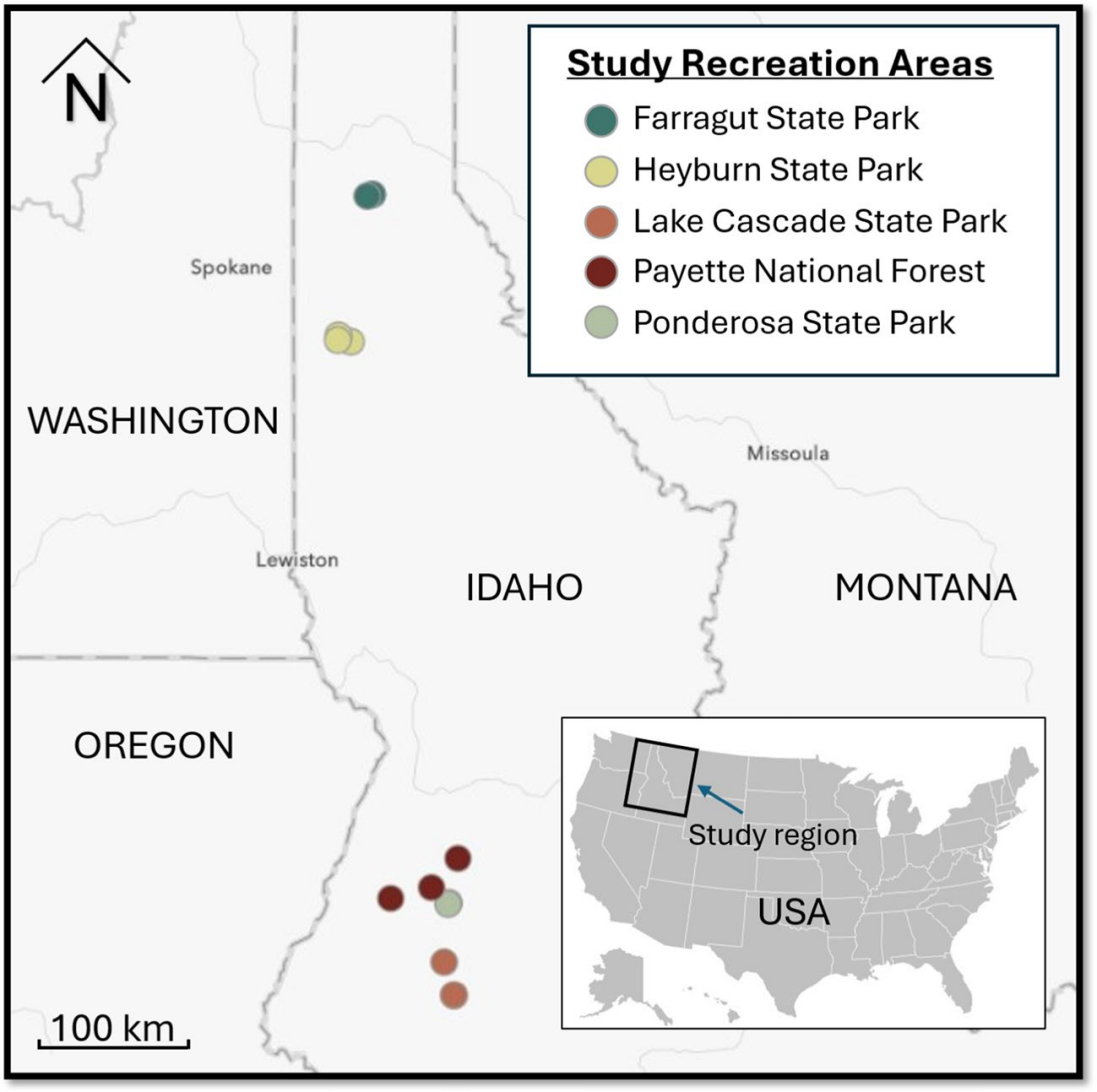
Study locations across Northern Idaho

For this study, litter sampling sites were selected in four state parks and one national forest (Fig. 1). Recreational areas were selected based on accessibility to the research team and the management’s willingness to provide the researchers with access to their campgrounds and camp hosts. In all cases, camp host involvement in the research process was at the discretion of the individual camp hosts. The campgrounds sampled within each recreation area were selected in consultation with recreation area managers. We specifically were interested in campgrounds that see significant usage during the prime camping season (May–October). Sampling at each location was stratified by campground, and specific campsite sampling locations were selected using a random number generator (*n* = 3 per campground).

Day use areas, recreational spaces open to visitors during the day without overnight accommodation, were sampled in three state parks (Farragut, Heyburn and Lake Cascade). In Ponderosa State Park day use areas are all lake-front beaches. The dominant wind pattern in the area drives lake debris up on the shorelines making determination of litter origin difficult. For this reason, we did not sample a day use area in this park. No day use area was surveyed in the national forest. The day use areas selected for inclusion in the study were located close to, though not within, our selected study campgrounds and were distinct from other recreational amenities such as boat ramps or group shelters.

Trash receptacles are provided in the state park campgrounds but are not provided in the national forest campgrounds. Trash receptacles in state park campgrounds are shared use and generally consist of dumpsters or bins located near the campground entrance and near the park restrooms. There are no trash receptacles provided at individual campsites. Trash receptacles are provided in state park day use areas as well.

### Macrolitter collection and categorization

Our litter collection and categorization methods were developed based on the Keep American Beautiful (KAB) 2009 national roadside litter survey, which developed a comprehensive list of litter types from multiple prior studies conducted worldwide (Schultz et al., 2009). Modifications were made to the classification categories based on differences in sampling locations (campgrounds vs roadsides). Our methods also closely align with the Environmental Protection Agency’s Escaped Trash Assessment Protocol (EPA, 2021), which was published just as we started this collection work.

Sample collections bracketed the summer camping period in Northern Idaho, which generally runs from the last week of May through the first week of October. During that period, samples were collected from each campsite sampling location by either the camp hosts (CH), the research team (RT), or both.

**Fig. 2:**
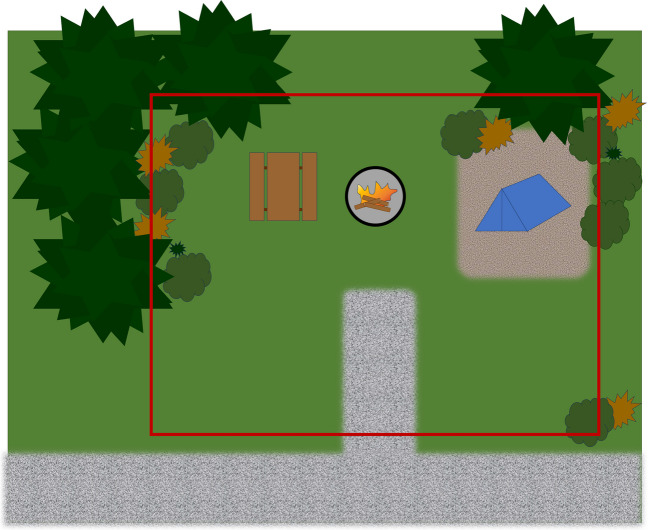
Typical campsite litter collection area. The red bounding box indicates the flagged litter collection area

For each of the campsite sample locations, a rectangular area including the fire pit, the tent pads, part of the driveway, and parts of the vegetated outlying area was defined and flagged for repeat sampling (Fig. 2). The designated area was measured with a meter tape with a precision of ± 0.1 m^2^ to determine a total sample area in meters for each campsite (average 219.0 m^2^ ± 10.6 m^2^).

The sampling area for day use sites focused on high-use areas within the day use regions. Sampling in these areas centered on a designated seating area (whether the site was a beach or a picnic area) and extended into the surrounding region to ensure the inclusion of other day use activities (shade shelters, lawn/beach games, etc.). Day use areas were defined and flagged like study campsites for repeat sampling and area determination (average area 362.7 m^2^ ± 38.7 m^2^).

Litter collection within flagged regions occurred in two ways. At the beginning of the camping season and every two to three weeks throughout the season, the RT visited the sampling sites in person. During these visits, the team completed a thorough litter collection of all visible litter (>0.5 cm) within pre-flagged sampling footprints (campsite and day use) to retrieve any litter not already collected by campers or CH. Access to sampling sites depended on campsite occupancy; if occupied the team secured explicit permission from the occupants before collecting any litter. If occupied and campers were not present or were present but did not give collection permission to the RT, no collections occurred. Most sites within the study were successfully sampled by the RT three times during the camping season. Five of the sites were sampled only twice by the RT.

Second, CH collected litter for the study as part of their normal cleaning duties at each study site. The RT met individually with CH to provide collection and storage instructions. All litter normally collected by CH as part of their campsite turnover cleaning routine within the research footprint was retained and stored in labeled paper bags for the RT. For safety and hygiene purposes, the CH were instructed not to save any biodegradable or hazardous waste items and instead to dispose of such waste as required by the recreation area waste management policy. Camp hosts stored litter samples from each separate cleaning event in a waterproof bin until they were retrieved by the RT. All cleaning, sorting, and categorization of collected litter were done by the RT.

State park day use areas within our study did not have regular cleaning schedules by either park staff or volunteers, though park staff did irregular cleanups depending on time and availability. No litter was saved during these irregular cleanups. For this study, the RT sampled each day use area three times during the summer camping season.

All litter (both RT and CH collected) was returned to the lab for cleaning, categorization, and analysis by the RT. Most litter pieces were covered in soil due to exposure to natural conditions, necessitating cleaning before determining litter weight. Where possible, litter pieces were washed with tap water and air dried for 24 h at room temperature before categorization. The few exceptions to the washing protocol were items that were partially or extensively burned, plastic-coated paper, and fragile pieces at risk of further fragmentation. For these samples, any attached debris was gently removed using a soft-bristle brush. Care was taken to protect the stored litter from any impact that could create post-collection fragmentation.

**Fig. 3:**
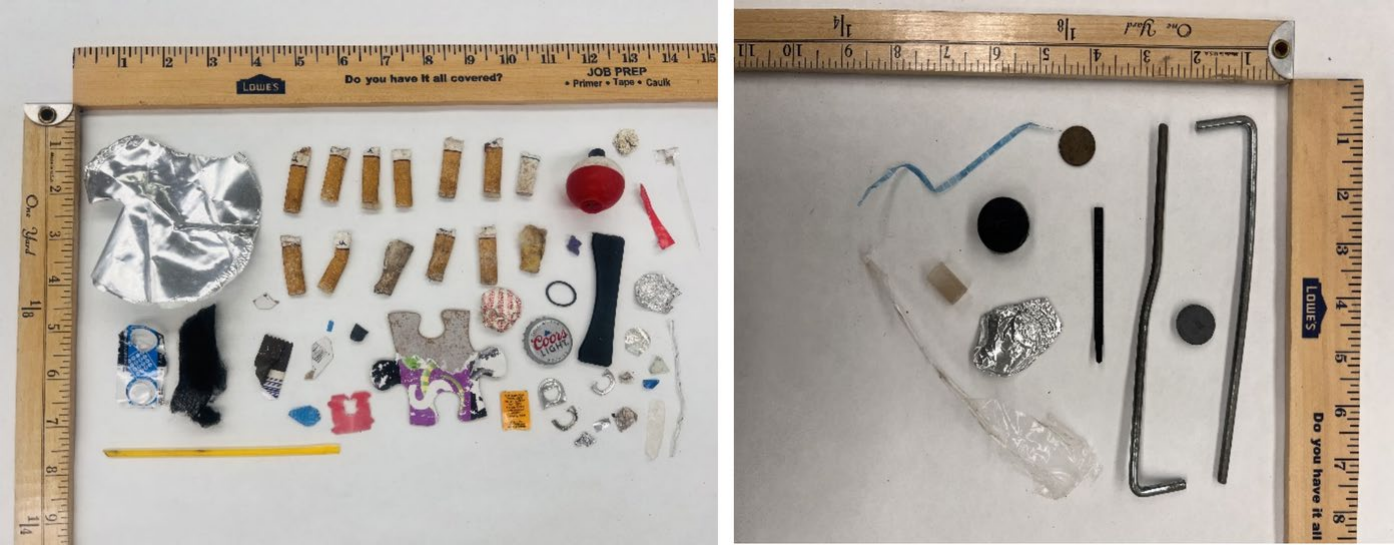
Examples of litter collections. Left: Van Wyck (A6) Research Team collection on June 23, 2021. Right: Last Chance Campground (10) camp host collection on August 3, 2021

After cleaning and drying, litter pieces from each collection (CH or RT) were photographed (e.g., Figure 3) and sorted into material classes (plastic, metal, glass, rubber, and mixed) and litter type before counting and weighing each class/type subgroup. The mixed litter class contained items whose exact material could not be determined, as well as litter that was a mixture of two or more classes, e.g., twist ties for bags with a thin metal wire covered by plastic. Within each material class except mixed, a miscellaneous sub-type included litter that did not fall explicitly within the other sub-type for that material class. If a whole piece of litter was found fragmented and divided into multiple pieces, each piece was counted as one piece of litter. The aggregate dry weight of all litter items within each sub-type and collection (CH or RT) was determined to the nearest 0.0001 g on an electronic balance.

### Litter analyses

For analysis, we defined litter collected by CH and diverted into established waste streams through routine cleaning as managed litter. Litter collected by the RT then represents unmanaged litter or waste that escaped CH cleaning actions and entered the environment.

The most common waste materials classes and types were determined by calculating a proportional mean percentage across all surveys, following the example of McGruer et al. (2025). To do this we calculated the percentage in each material class or material type for each survey and then averaged individual survey percentages across all surveys. Confidence intervals (95%) were estimated using bootstrapping with replacement (10,000) of the mean values.

Explanatory variables collected at the campsite level include number of visitors, occupancy rate, visitor population density, and the provision of trash receptacles. We did not include campsite type as an explanatory variable as we did not have data about the actual amenity use by visitors (e.g. some campers use tents in sites with electrical hookups). See supplementary file 1 and 2 for site-specific data including area, occupancy rates, number of visitors, and the presence or absence of trash receptacles, etc.

Campground reservation data provided by the recreation area managers allowed us to calculate the campsite occupancy rate (percentage of days occupied) over the 100-day camping season (between the last Monday in May and the first Monday in September). From the occupancy rate during the camping season, we estimated the number of occupied days during the research duration (time between the first research collection and the last research collection), as these data were not available for some locations. The occupancy and visitor data for Payette National Forest cannot be broken down on a campsite basis and are instead presented at the campground level. The total occupied days do not consider the number of total turnovers during the season.

Visitor population density was calculated as the number of daily visitors (total visitors during the camping season divided by the number of days in the camping season) divided by the research area in km^2^ to give visitors per km^2^. Population density could not be determined for day use areas as no data were available on the number of day use area users during the camping season.

### Litter density

The number and mass of the items collected at each campsite by both the CH and the RT were used to calculate litter densities by count and mass as items/m^2^ and g/m^2^. Campsite areas were measured with a field tape with a precision of ± 0.1m^2^. When scaled to km^2^ for comparison with published literature, measurement error propagates to ± 100 m^2^/km^2^, which remains small relative to standard errors from sampling variability. Reported uncertainties primarily reflect natural variation in litter accumulation patterns rather than measurement error.

To discuss spatial differences in litter densities, we looked at differences between campsites within the same campgrounds, differences between campgrounds within the same recreation area and differences between recreation areas. To discuss temporal differences in litter densities, the sampling interval between surveys was determined.

### Accumulation rates

Repeated sampling in the same location allowed for the calculation of accumulation rates by both number and mass. To measure litter accumulation rates, we established a baseline by removing all litter during our initial RT visit to each campsite. We then calculated managed and unmanaged litter accumulation as the litter collected during each subsequent CH or RT survey, making the surveys the base statistical unit for accumulation rate calculations. These accumulation rates were expressed for each campsite in items/km^2^/day and kg/km^2^/day, by dividing the count or mass of items collected by the area surveyed and the number of days between sampling events. Because each RT visit returned the site to a clean state, we treated each survey as an independent measure of litter accumulation between visits for the RT data. We could not make the same assumption regarding the CH litter accumulation rates and so those data are not used for statistical analyses of accumulation rates.

Using the number of campsites, median accumulation rates for unmanaged litter, average campsite area, and average occupancy rate, we estimated the total uncontrolled litter on a per-campground basis during the camping season. We chose the median accumulation rate as a representative value because the underlying data are heavily skewed toward low accumulation rates with a few extreme outliers. We calculated total comparable area by multiplying average campsite area by the number of campsites, since these areas experience similar use intensity and are likely to have similar litter accumulation rates. We did not extend this analysis to the whole campground area (roads, bathrooms, space between campsites, etc.) or to the day use areas because the use intensity across those areas was not similar.

### Statistical analysis

To assess the factors influencing the spatial and temporal variations of litter and comparisons between the characteristics of the sites (occupancy rate, number of visitors, campground, recreational area, and provision of trash receptacles) and the densities of litter (by count and by mass) we evaluated the data using the Kruskal–Wallis with Dunn’s post-hoc tests and Mann–Whitney *U* tests. We identified potential outliers using Mahalanobis distance but retained all data points after verification revealed they represented valid observations rather than measurement or data entry errors. These comparisons were considered significant if *p*-values were equal to or lower than 0.05. These statistical parameters were calculated using the “tidyverse” package (Wickham et al., 2019) in RStudio version 2024.09.1.394 (RStudio Team 2024). Both statistical analyses are non-parametric as our data does not show a normal distribution and contains outliers.

### Camp host impacts

To estimate potential CH impact across the campgrounds in our study, we calculated an average CH litter removal rate for each campground (total litter collected by the CH from all survey sites within one campground divided by the number of campsites surveyed times the number of days in the research period). Multiplying this rate by the total number of campsites within each campground and the number of camping season days (100 days) allows us to estimate the number of litter pieces collected by the CH from all campsites in each campground during the prime camping season.

To estimate the broader impact of CH programs, we applied our calculated average CH litter collection rate to the total number of campsites served by CH across Idaho State Parks and federal lands. Idaho State Parks system campsite numbers were obtained through personal communication with park management (R. Honsinger, October 28, 2024). Information on federal recreation area campsite numbers was obtained from Bureau of Land Management and US Forest Service sources (BLM, n.d.; S. Bassista, personal communication, November 11, 2024). Estimated annual litter removal across public land types was determined by multiplying the average daily collection rate from this study by the number of hosted campsites and the 100-day camping season. Total mass was calculated using the overall proportional mean litter mass per piece from this study.

We estimated the economic value of CH litter removal services using inflation-adjusted cost estimates from Stein (2005). Cleanup costs were calculated as $0.26 per item for volunteer labor and $1.85 per item for paid labor (adjusted to 2021 dollars using the Consumer Price Index). Total cost savings were calculated as the difference between paid labor costs and volunteer labor costs for all litter items removed by CH during the study period. Per-site costs were calculated by dividing total costs by the number of campsites in the 12 campgrounds with camp host data. To estimate statewide savings, we extrapolated our per-site cost savings to Idaho’s 27 state park campgrounds (approximately 1,950 sites total).

## Results

Table 1 shows the study locations, sampling period, and specific campsites sampled for this study. The sampled sites ranged in size from 113.4 to 434.3 m^2^ with an average area of 219.0 m^2^ ± 10.6 m^2^. A total of 4834 litter items, weighing 13,447.1 g (29.7 lbs.), were collected from all sites during the study period of May through October 2021. Four thousand three hundred fifty-one (90%, 10.2 kg) of those items were collected in campgrounds, and 483 items (10%, 3.3 kg) came from day use areas. Within campgrounds, the RT recovered 2252 litter items (51.8%), and the CH collected the remainder (2099, 48.2%). The RT collected all the litter from day use areas. Supplementary file 1 and 2 give the breakdown of site areas and litter by classes and types in individual campgrounds and day use areas.

**Table 1:** Sampling information by recreational area, including research period, campground or day use area name, sampling sites, number of surveys collected by the RT and CH at each campsite, and the type of campsite (Tent is for tent campers only, Basic is for either tent camper or RV campers, Electric is a Basic site with electricity provided for RV campers, Day Use areas have no overnight camping allowed)

Recreation areas (research period)	Campground or *day use area* Name	Site # (RT/CH) Site type	Site # (RT/CH) Site type	Site # (RA/CH) Site type
Heyburn State Park (May 26–Sept 25)	Hawley’s Landing	2 (3/9) Tent	7 (3/9) Tent	18 (4/8) Basic
	Chatcolet	102* (4/0) Basic	113 (4/10) Basic	119 (4/10) Basic
	Benewah	205 (4/17) Basic	217 (4/15) Basic	225 (4/13) Tent
	*Plummer’s Point*	1 (3/0) Day Use*		
Farragut State Park (May 27–Sept 25)	Waldron	156 (4/22) Electric	180 (3/7) Electric	200 (4/16) Electric
	Snowberry	106 (3/15) Electric	121 (4/12) Electric	134 (3/5) Electric
	Whitetail	7 (4, 11) Basic	42* (3/0) Basic	49 (2/2) Basic
	*Beaver Bay*	1* (3/0) Day Use		
Ponderosa State Park (Jun 24–Oct 3)	RV	201 (3/2) Electric	234 (2/5) Electric	236 (2/7) Electric
	Peninsula	2* (3/0) Electric	17* (3/0) Electric	72* (3/0) Electric
Lake Cascade State Park (Jun 24–Oct 3)	Poison Creek	241 (3/3) Electric	242 (3/6) Electric	247 (4/4) Electric
	Van Wyck	A6 (3/4) Basic	C4 (3/3) Basic	D5 (4/3) Basic
	Ridgeview	183 (3/4) Electric	185 (3/6) Electric	191 (4/6) Electric
	*Van Wyck*	1* (3/0) Day Use		
Payette National Forest (Jun 24–Oct 3)	Cold Springs	10* (4/0) Basic	19* (3/0) Basic	27* (4/0) Basic
	Last Chance	2 (3/10) Basic	10 (3/8) Basic	16 (3/9) Basic
	Upper Payette	12 (2/5) Basic	14 (3/2) Basic	18 (2/2) Basic

*Sites with no CH collection

### Litter composition

Plastic-dominated litter composition across surveys, representing 78.6% (95% CI: 72.1–85.3%) of items collected and 65.6% (95% CI: 62.0–69.1%) of total mass. Metal was the second most common material at 14.7% (95% CI: 12.8–16.7%) by count and 23.2% (95% CI: 20.0–26.4%) by mass. Glass (1.2%, 95% CI: 0.6–2.0%), rubber (3.0%, 95% CI: 1.8–4.5%), and mixed items (2.5%, 95% CI: 1.5–3.7%) each represented 3% or less by count but contributed 2.6%, 4.5%, and 4.0% of total mass, respectively (Table 2).

**Table 2:** Litter by material class and type, including total litter number, total litter mass, proportional mean litter mass per piece, proportional litter percentage and proportional litter mass percentage with 95% confidence intervals determined by bootstrapping. These categories represent the survey categories used to tally litter data

Item(s) by class and type	Litter (#)	Litter mass [g]	Mean litter mass per piece (95% CI) [g]	Litter (95% CI) [%]	Litter mass (95% CI) [%]
Plastic					
Food wrappers	729	252.1	0.35 (0.23–0.49)	28.5 (25.5–31.5)	15.2 (11.8–19.0)
Beverage bottles	25	414.9	19.05 (11.46–28.18)	14.9 (10.1–20.1)	52.1 (40.8–62.5)
Other plastic containers	19	323.7	19.59 (9.18–33.54)	16.3 (7.8–28.8)	33.5 (21.6–46.9)
Container lids/caps	154	159.9	1.09 (0.92–1.29)	15.0 (12.5–17.7)	17.3 (13.3–21.7)
Cigarette butts	572	207.3	0.27 (0.23–0.32)	27.7 (22.9–33.0)	17.5 (12.1–23.3)
Plastic rope	18	184.9	10.86 (2.89–24.67)	8.8 (5.5–12.9)	24.5 (12.9–37.0)
Fishing line and lures	5	14.1	3.49 (0.10–10.04)	24.4 (3.8–45.0)	28.8 (0.3–74.0)
Polystyrene	148	27.8	0.29 (0.16–0.46)	16.0 (12.4–19.8)	4.4 (2.1–7.3)
Plastic utensils	25	81.8	3.60 (2.90–4.27)	12.7 (7.3–19.5)	25.4 (14.5–38.4)
Plastic straws	48	22.3	0.50 (0.40–0.61)	10.7 (8.4–13.2)	8.7 (4.7–13.7)
Syn. clothing material	115	912.5	4.60 (2.20–7.71)	15.4 (12.7–18.5)	17.7 (12.3–23.5)
Personal care products	55	78.4	1.46 (0.52–3.05)	14.3 (9.7–19.9)	13.4 (6.6–21.7)
Plastic tarp	69	2.53	0.08 (0.01–0.19)	16.6 (11.5–22.4)	1.6 (0.5–3.1)
Plastic balloons	7	1.81	0.45 (0.01–0.89)	11.8 (1.9–25.8)	1.0 (0.1–2.7)
Plastic-coated cardboard	281	679.3	2.69 (1.33–4.46)	22.0 (18.8–25.5)	18.4 (14.4–22.8)
MISC. plastic pieces	1535	1807.9	1.66 (1.23–2.23)	42.2 (39.6–44.9)	37.7 (34.1–41.4)
Total plastic	3805	5171.3	1.35 (1.10–1.61)	78.6 (76.4–80.8)	65.6 (62.0–69.1)
Metal					
Aluminum/tin foil	289	140.4	0.61 (0.35–0.97)	18.7 (16.1–21.5)	13.4 (9.7–17.4)
Aluminum/tin container	27	235.6	9.80 (6.53–13.29)	11.5 (7.3–16.0)	34.0 (21.3–46.7)
Bottle caps	113	227.8	1.99 (1.89–2.08)	15.0 (10.8–20.3)	31.1 (24.0–38.5)
Can tabs	62	36.6	0.63 (0.40–0.92)	12.6 (9.2–16.3)	8.5 (4.9–12.4)
Beverage cans	26	382.1	14.41 (13.35–15.58)	9.5 (7.0–12.1)	41.0 (26.7–56.1)
Aerosol cans	9	169.8	27.18 (2.62–70.51)	15.7 (8.9–21.7)	50.3 (17.0–83.7)
MISC. metal pieces	158	2318.9	23.48 (7.56–47.14)	18.5 (14.4–22.9)	49.7 (41.6–57.5)
Total metal	684	3511.3	6.92 (3.07–12.69)	14.7 (12.8–16.7)	23.2 (20.1–26.4)
Glass					
Glass bottle	6	1172.1	233.5 (108.9–349.6)	4.9 (1.8–8.7)	60.3 (37.3–81.6)
Jar	1	10.6	10.56 (NA-NA)	11.1 (NA-NA)	65.8 (NA-NA)
MISC. glass pieces	92	276.4	3.44 (1.88–5.91)	13.4 (10.2–17.1)	21.6 (15.0–29.1)
Total glass	99	1459.1	24.48 (4.23–52.12)	1.2 (0.8–1.7)	2.6 (1.6–3.8)
Rubber					
Gloves	6	28.1	3.93 (1.29–6.80)	18.5 (6.4–39.6)	21.4 (8.4–43.3)
Latex balloons	2	3.59	1.80 (0.17–3.42)	9.2 (5.9–12.5)	7.5 (1.8–13.2)
MISC. rubber pieces	131	1646.6	19.54 (2.16–51.71)	14.7 (11.9–17.8)	22.4 (16.4–28.8)
Total rubber	139	1678.3	18.79 (2.29–49.60)	3.0 (2.2–3.9)	4.5 (3.1–6.1)
Mixed (combination items)					
Item(s)	107	1694.1	14.1 (11.1–17.6)	23.0 (16.5–29.9)	14.1 (11.1–17.6)
Total mixed	107	1694.1	2.5 (1.8–3.3)	4.0 (2.7–5.5)	2.5 (1.8–3.3)
Totals	4834	13,447.8	2.50 (1.70–3.81)	100	100

The average mass per litter piece across surveys was 2.50 g (confidence intervals are reported in Table 2 unless otherwise indicated), with substantial variation by material class. Plastic had the lowest average mass per piece at 1.35 g, while glass had the highest at 24.48 g. Metal averaged 6.92 g per piece. Rubber litter averaged 18.79 g per piece, though this was heavily influenced by a single survey containing a tire weighing 1186 g; excluding that survey, rubber pieces averaged 4.38 g.

Within material classes, miscellaneous (unidentifiable) items were prevalent: 42.2% of plastic, 18.5% of metal, 13.4% of glass, and 14.7% of rubber items. Among identifiable plastic items, food wrappers (28.5%) and cigarette butts (27.7%) were most common. Aluminum foil was the most abundant identifiable metal item (18.7%) and gloves were most common among rubber items (18.5%), though with high uncertainty due to their rarity (6 items total).

Overall, the four most common identifiable litter types were plastic food wrappers (15.1%, 729 items), cigarette butts (11.8%, 572 items), aluminum foil (6.0%, 289 items), and plastic-coated cardboard (5.8%, 281 items). No other identifiable type exceeded 5% of total litter composition by count.

By mass, miscellaneous items across all material classes collectively represented 57.3% of total litter weight (7,743 g). Among identifiable items, glass bottles (8.7%, 1172 g), synthetic clothing (6.8%, 913 g), and plastic-coated cardboard (5.1%, 679 g) were the top three contributors by mass, with no other identifiable type exceeding 5%.

### Litter by location

The pattern of material classes is very similar across all recreational area locations with plastic being the dominant class everywhere and accounting for a proportional average of 74.0 (95% CI: 69.4–78.5%) to 80.7% (95% CI: 76.1–85.1%) of all litter across locations and site types (campground or day use). Metal was the next most common litter material ranging from an average of 13.4 (95% CI: 9.4–17.8%) to 18.9% (95% CI: 14.6–23.4%) across all surveys by location. All other material types were under 4% by location.

**Fig. 4:**
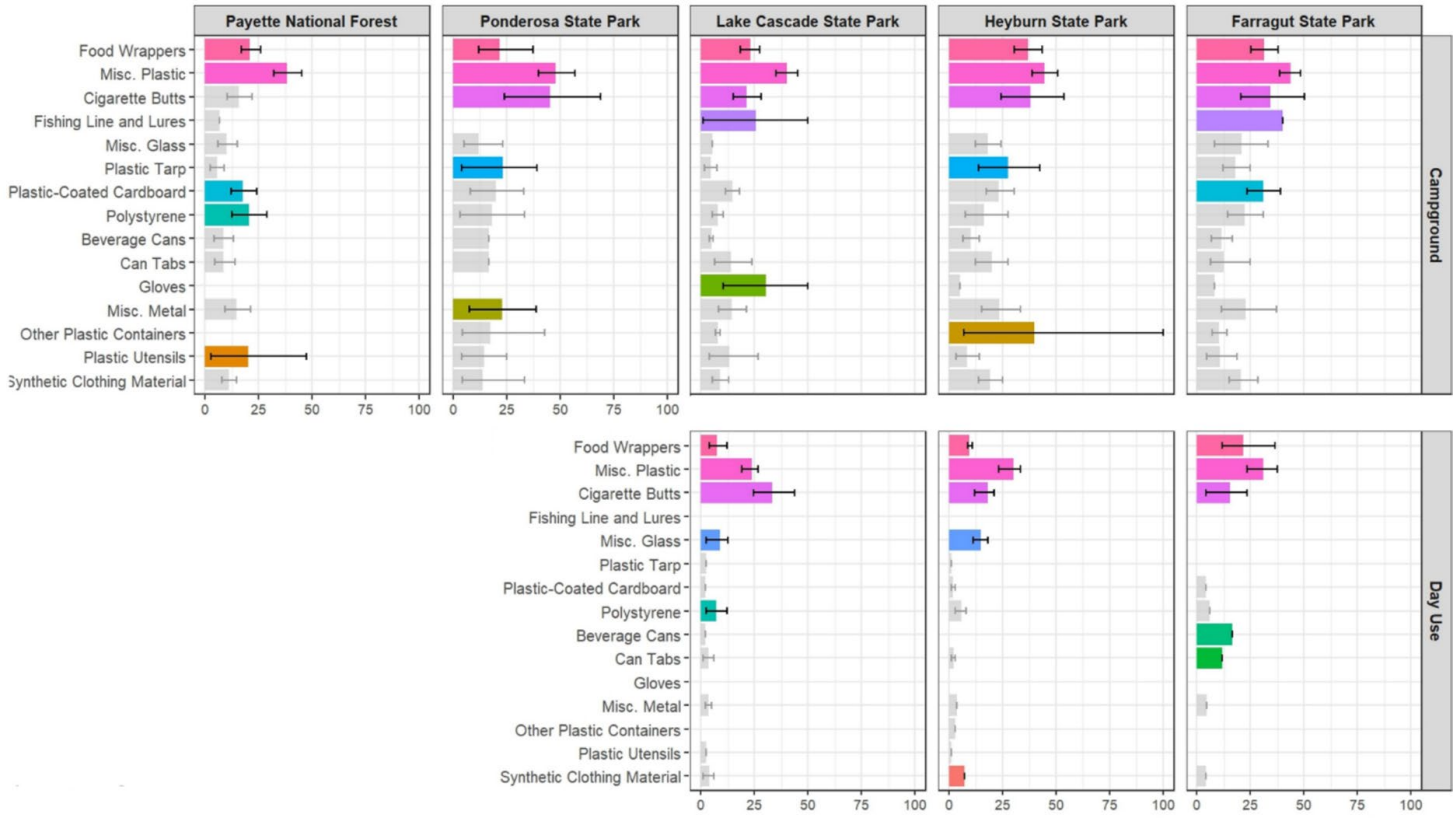
Colored bars represent the top 5 types of litter collected over the research period plotted by proportional mean percentage with 95% CI and use type (Campground and Day Use) for each recreation area. Grey bars indicate the amount of litter found within a litter type which is not in the top 5 types of litter. For visual clarity, litter types that were not in the top 5 in any area are not displayed. We sampled two campsites within each of three campgrounds at Ponderosa State Park and three campsites within each of three campgrounds in all other recreation areas (total *n* = 42 campsites, *n* = 14 campgrounds). Day use areas the three state parks (Lake Cascade, Heyburn, and Farragut) each had one sampling site

The types of litter (Fig. 4) by proportional mean percentages reveal some similarities and differences across locations and use types. In campgrounds, food wrappers and miscellaneous plastic are dominant litter types across all locations, and cigarette butts are top five in all state park locations, though not in Payette National Forest. No other litter type in the top five occurs in more than two locations. Day use area patterns are similar to those seen in campgrounds, with food wrappers, miscellaneous plastic and cigarette butts in the top five in all areas.

### Litter density and litter accumulation rate

Litter densities were highly variable by site, with managed litter density (CH collections) by count ranging from 2796 to 338,346 items/km^2^ (0.029–5411 kg/km^2^ by mass) and unmanaged litter density (RT collections) by count ranging from 6974 to 272,000 items/km^2^ (0.345–6593 kg/km^2^ by mass). The median litter density was 25,121 items/km^2^ and 51,238 items/km^2^ (11.6 kg/km^2^ and 107.7 kg/km^2^) in the managed and unmanaged litter collections respectively.

Managed litter accumulation rates range from 118 to 12,364 items/km^2^/day (median 4024 items/km^2^/day) and from 0.006 to 300 kg/km^2^/day (median 2.13 kg/km^2^/day). Sampling intervals ranged from 1 to 47 days (median 6 days). Unmanaged litter accumulation rates ranged from 165 to 309,218 items/km^2^/day (median 1250 items/km^2^/day) and from 0.002 to 773 kg/km^2^/day (median 2.18 kg/km^2^/day). Sampling intervals ranged from 21 to 122 days (median 43 days). We found a significant negative relationship between sampling interval and accumulation rate for both CH (*r* = −0.21, *p* < 0.001, *n* = 270) and RT (*r* = −0.26, *p* < 0.01, *n* = 101) collections, with shorter intervals associated with higher daily accumulation rates.

Using median accumulation rates for unmanaged litter in each campground, we estimated the flux of litter into the environment (Supplemental Table 1). We chose the median accumulation rate as a representative value because the underlying data are heavily skewed toward low accumulation rates with a few extreme outliers. This calculation determined that despite camp host intervention, approximately 27,757 pieces entered the environment for 14 of the campgrounds (20,367 pieces for 12 of the campgrounds that also have host collection) during the 2021 camping season.

### Controls on litter

We tested the importance of several explanatory variables (day of the week, location, provision of trash receptacles, and use type) to better understand controls on litter in these sites. Because there is a large variability in the frequency of CH collection, the managed litter collection was not analyzed in relation to external controls. The following analyses were performed only on the clean unmanaged litter dataset.

Kruskal–Wallis tests indicate significant differences across days of the week in the litter count density, count accumulation rate, mass density and mass accumulation rate (Table 3), except by count density when day use areas are included. However, further pairwise post-hoc comparisons using a Dunn test with a Bonferroni adjustment indicate no significant differences between days of the week for count density, count accumulation rate, or mass accumulation rate. The only remaining post-hoc significance is the difference between mass density of litter collected on Sundays and Wednesdays both with (*p* = 0.038) and without (*p* = 0.013) day use areas.

**Table 3:** Kruskal–Wallis Rank Sum Test for difference by collection day of the week. While Kruskal–Wallis results indicated significance across all measures, only mass density retained significance after a pairwise post-hoc Dunn test with a Bonferroni adjustment

Test metric	With day use area			Without day use area		
	*H*	*df*	*p*	*H*	*df*	*p*
Count Density	9.3398	4	0.053	10.428	4	0.034
Count Accumulation rate	15.407	4	0.004	15.038	4	0.005
Mass density*	14.681	5	0.005	15.92	4	0.003
Mass accumulation rate	9.6138	4	0.048	10.662	4	0.031

* indicates significant results at the 95% confidence interval

Kruskal–Wallis tests indicate no significant differences between locations by litter count or mass density or accumulation rates (Table 4). This was true with both inclusion and exclusion of day use areas.

**Table 4:** Kruskal–Wallis Rank Sum Test for difference by recreational area location

Test metric	With day use area			Without day use area		
	H	df	*p*	H	df	*p*
Count density	5.1663	4	0.271	5.5852	4	0.232
Count accumulation rate	7.6105	4	0.107	7.1517	4	0.128
Mass density	2.3786	4	0.667	2.3382	4	0.674
Mass accumulation rate	4.034	4	0.401	3.7614	4	0.439

Similarly, Mann–Whitney *U* tests indicate no differences between sites with trash receptacles and those without in litter count or mass density or accumulation rates, with or without the inclusion of day use areas (Table 5).

**Table 5:** Mann–Whitney *U* tests of the importance trash receptacles on the amount of litter (trash receptacles vs none)

Test metrics	With day use area		Without day use area	
	*U*	*p*	*U*	*p*
Count density	928	0.109	886	0.068
Count accumulation rate	883	0.229	839	0.167
Mass density	858	0.327	823	0.219
Mass accumulation rate	854	0.345	820	0.230

However, there are significant differences in litter density and litter accumulation rate by mass, though not by count, between day use and camping sites (Table 6).

**Table 6:** Mann–Whitney *U* test for difference by site use (day use vs camping area)

Test metric	*U*	*p*
Count density	152	0.057
Count accumulation rate	178	0.126
Mass density*	148	0.050
Mass accumulation rate*	144	0.041

* indicates significant results at the 95% confidence interval

### Camp host impact

Camp hosts collected litter from study campsites in 12 of 14 campgrounds and 32 of 42 campsites (Table 1). CH did not collect any litter from day use areas. CH collected a total of 2,099 pieces of litter which represents 46.6% of all litter collected in this study. When day use areas are removed the percentage of litter collected by CH rises to 51.7%. At individual campsites, CH litter collection accounted for between 10.8 and 79.6% of all litter collected. We estimate that CH are responsible for removing, at a minimum, 25,796 pieces of litter across the 12 hosted campgrounds in our study (Supplemental Table 2). Using an average litter weight of 2.50 g, this represents 64.5 kg of litter during the season.

Based on our calculated average camp host litter collection rate of 0.57 pieces per campsite per day (range 0.19–1.06 pieces per day) and Idaho State Parks’ 1,950 campsites served by camp hosts (R. Honsinger, personal communication, October 28, 2024), we estimate that camp hosts remove upwards of 111,150 pieces of litter from Idaho State Parks annually over the 100-day camping season. At an average weight of 2.50 g per piece, this represents more than 277 kg (611 lbs) of litter removed each season across the state by camp hosts alone. This should be considered a conservative estimate as not all litter collected by camp hosts was retained for this study. Though federal recreation areas within Idaho are also served by camp hosts, we could not estimate the CH impact in those areas because the number of campsites served by CHs in 2021 could not be verified.

Based on litter removed across the 12 campgrounds with CH data in this study, volunteer camp host cleanup efforts represented a cost savings of $41,015 (the difference between $47,722 in estimated paid labor costs and $6,707 in volunteer labor costs). Per campsite, this translates to approximately $14 per site in volunteer camp host costs versus $102 paid labor costs over the 2021 camping season. Extrapolating to Idaho’s 27 State Parks campgrounds (approximately 1,950 sites), volunteer camp hosts potentially saved the state more than $171,600 in litter cleanup costs during the 2021 camping season alone.

## Discussion

This study presents the first data on campground litter abundance and composition in Idaho. Accumulation of litter (both plastic and non-plastic) debris on land is simultaneously one of the most ignored and the most visible emerging environmental issues (KAB, 2021). The results of this study highlight litter and littering in our state parks and national forests and adds to our understanding of the characteristics and accumulation rates of litter in terrestrial systems. We also highlight the impact of CH and their importance in properly managing litter in recreational settings.

### What kinds of litter are left behind?

Litter in our recreational areas has some interesting differences from litter collected in other settings. On average across surveys, plastic makes up 78.6% (95% CI: 72.1–85.3%) of litter counted and 65.6% (95% CI: 62.0–69.1%) of the litter mass. While direct comparison with studies using different analytical approaches (direct vs proportional means) should be made cautiously, the magnitude of difference suggests important patterns. The average proportion of plastic by count in this study is more than 35% higher than litter found along roadways or waterways by KAB (KAB, 2021) and in state and county parks in the Washington State litter survey (Washington Department of Ecology, 2023). This is also close to the high end of the range found by Ansari and Farzadkia (2022) when they reviewed studies of ocean beaches (11–99.8%). The average proportion of plastic by mass (65.6%) is also much higher than the weight percentage of plastic found in traditional waste streams globally (7–17%) (Eurostat, 2007; US EPA, 2006; Barlaz, 2006; Burnley, 2007; Sokka et al., 2007). This finding is even more striking because in other places with a high amount of plastic litter there is usually a physical mechanism to separate and concentrate the plastic litter (ocean beaches, waterways, wind exposure, etc.). That was not the case in this study, and we intentionally excluded a survey site (Ponderosa Park day use area) where we were not confident in our ability to distinguish between visitor-imported litter and lake/wind-deposited litter. In addition, the frequency of cleanups in recreation areas is far higher than in most other natural areas, meaning much of the litter is recaptured before it has a chance to disperse or fragment due to weathering. This suggests that the composition of litter found in these areas closely reflects the actual composition of materials coming into recreational areas.

The predominance of single-use plastics likely explains the high plastic content observed in recreational litter compared to traditional waste streams. Plastics are widely used for primary food and beverage packaging (Operato et al., 2025) and plastic packaging makes up nearly half of all plastic waste produced globally (Phelan et al., 2022). In our study, miscellaneous plastic makes up 31.8% of all litter found as a direct percentage, and across surveys miscellaneous plastic averages 42.2% (95% CI: 39.6–44.9%) of litter found (Table 2). This plastic is not identifiable to source, but many of the most recognizable types of litter are single-use plastics. Food wrappers and cigarette butts are very common across all locations and use types, and plastic-coated cardboard, polystyrene (e.g. beverage cups), plastic utensils, and other plastic containers all occur in the top five types of litter in various places in this study (Table 2, Fig. 4). These plastic types are all generally associated with food and beverage consumption or smoking, suggesting that consumption is a primary driver of litter in these recreation and day use areas. Importantly, consistency in the dominant types of litter across all recreation areas and uses (camping vs day use), suggests that education and mitigation efforts focused on single-use plastics and consumption-related litter types would have broad applicability.

### Factors influencing spatial variability

The population densities experienced in these campgrounds over the camping season (median population density 4252 people/km^2^, min/max 1110/14,285 people/km^2^) are similar to population densities found in many major urban areas. This contrasts sharply with the population densities of the counties surrounding the recreation areas, which the 2020 Census data show as having population densities of 145 people/km^2^ or less (U.S. Census Bureau, 2020). This suggests that these popular, heavily used camping areas are experiencing much greater litter and waste management pressures than would be expected based on their perception as “wild” spaces.

Given these urban-level population densities, comparing our data to urban litter data is reasonable. However, median litter densities for our two collections (managed = 25,121 items/km^2^ and unmanaged = 51,238 items/km^2^) fall at the low end of litter densities found across a variety of urban settings globally (Ledieu et al., 2022, range = 30,000–14,270,000 item/km^2^; Schuyler et al., 2021, range = 40,000–580,000 items/km^2^). Several factors may explain why litter densities are low despite high visitor densities.

First, the sources and types of litter input differ between recreation areas and urban environments. Within recreation areas, there is typically little to no commercial activity apart from at the park headquarters (which were excluded from this study), and commercial sites generate substantially more litter than other urban land uses (Gholami et al., 2020; Keep Britain Tidy, 2020; Pietz et al., 2021; Xiong et al., 2022; Youngblood et al., 2022). Second, the physical environment of recreation areas constrains litter transport and accumulation differently than urban settings. Urban areas feature impervious surfaces, built infrastructure, stormwater conveyance, and wind corridors that facilitate litter transport and redistribution, often beyond the reach of regular cleanup efforts. In contrast, campgrounds tend to have vegetated terrain, tree canopy, irregular topography, and natural drainage patterns that trap and retain litter at deposition sites where it is more likely to be controlled.

Also, explicit regulations and social norms promote litter control in recreation areas. State park and national forest campground regulations require visitors to keep campsites clean and remove all litter before departure (Idaho Department of Parks and Recreation, 2022). While not always enforced, these rules are typically disseminated and reinforced by CH. Compliance is further motivated by the fact that campsite cleanliness is a known factor impacting camper satisfaction (Lee et al., 2019; Mikulić et al., 2017; Severt & Fjelstul, 2015), creating social pressure to maintain campsite cleanliness. Finally, active management interventions create a positive feedback loop that sustains low litter levels. CH conduct regular campsite cleaning, maintaining high levels of cleanliness. This is particularly important because research shows that littering occurs less frequently in areas with little existing litter (Kallgren et al., 2000; KAB, 2009). Together, these factors are likely to maintain lower litter densities in recreation areas compared to urban settings with similar population densities.

Litter densities in camping areas (median = 32,293 items/km^2^) were significantly lower than in day use areas (median = 94,400 items/km^2^), likely reflecting both greater population density in day use areas and higher cleaning frequency in camping areas.

Differences between managed and unmanaged collections also reveal important patterns. The RT collected significantly more and heavier litter per survey than CH (*U* = 633, *p* < 0.001 for number; *U* = 566, *p* < 0.001 for weight), despite CH collecting nearly twice as many surveys (270 vs 146). This likely reflects differences in collection approach and spatial focus. Lightweight plastic items, which dominate these datasets, are easily redistributed by wind into vegetation buffers (Khoeriyah & Sembiring, 2023). The RT explicitly searched the entire flagged footprint, including vegetation buffers. Litter in these areas is more difficult to see and access and therefore may have been missed during CH cleaning focused on making campsites visibly clean. In addition, the RT’s thorough search likely captured smaller cryptic items (such as plastic bread bag tags) that CH might miss during routine visual cleaning. The higher percentage of plastic collected by RT compared to CH (supplementary file 1 and 2) supports this interpretation.

### Factors influencing temporal variability

The rate at which litter accumulates in our recreation areas is highly variable. Managed litter accumulation rates ranged from 118 to 12,364 items/km^2^/day (median = 4024) and 0.006 to 300 kg/km^2^/day (median = 2.13). Unmanaged sites showed similar variability: 165 to 309,218 items/km^2^/day (median = 1,250) and 0.002 to 773 kg/km^2^/day (median = 2.18). Despite this variability, median accumulation rates were substantially lower than those reported for urban streets, urban streams, and other park settings (Cowger et al., 2022; Ledieu et al., 2024; Poletti & Landberg, 2021; Washington Department of Ecology, 2023).

Day-of-the-week also explains some of the temporal patterns in the data. While initial Kruskal–Wallis analysis shows significance across nearly all categories of count and mass density and accumulation rates (Table 3), only mass density remained significant after post-hoc Dunn test with Bonferroni adjustment. This indicates that there are significant differences in the mass density of litter collected between Sundays and Wednesdays, both with (*p* = 0.038) and without (*p* = 0.013) day use areas included. These findings suggest there are subtle, though real, differences between weekend and mid-week visitor littering behaviors, especially in campgrounds. However, these weekend/weekday differences do not appear to be a strong overall predictor of litter accumulation in these areas.

This study found a modest negative correlation between sampling interval and accumulation rate (*r* = −0.21 to −0.26), though sampling interval explained only 4–7% of variance in accumulation rates. This weak relationship likely reflects temporal variation in visitor use, with more frequent CH surveys conducted during peak use periods, and possible loss of ephemeral litter items between longer sampling intervals. However, most variation is attributable to other factors such as site characteristics, visitor density, and seasonal patterns.

Direct comparison of accumulation rates across studies is complicated by fundamental differences in sampling design and study settings. This study collected data on daily to monthly intervals in recreational areas with active management, while comparison studies (Cowger et al., 2022; Ledieu et al., 2024; Poletti & Landberg, 2021; Washington Department of Ecology, 2023) employed varying temporal designs (every few days to biannually) across different settings (highly urban to mixed urban-rural) with inconsistent accounting of cleaning activities. Each setting has different input rates from direct littering and imported litter, as well as different removal rates from deliberate cleaning and natural processes. Future studies would benefit from standardized methods that account for sampling interval (e.g., effort offsets, Bayesian hierarchical models), degree of urbanization, and cleaning frequency to improve comparability.

Since campgrounds lack permanent residential facilities and commercial activities, most litter inputs come from visitors who deliberately choose what to bring, potentially reducing litter diversity and volume compared to urban settings. Multiple overlapping management interventions such as signage, visitor education, and regular CH cleaning, work to reduce and properly dispose of litter. Together, these factors likely contribute to the low litter accumulation rates observed despite high population densities during prime camping season. This suggests that recreational settings may represent a distinct litter accumulation environment, characterized by relatively controlled inputs and active removal processes, compared to urban or unmanaged natural areas.

### Controls on litter and camp host impacts

Neither physical location nor presence of trash receptacles explains differences in litter density or accumulation rates (Tables 4 and 5). This held true both with and without the inclusion of day use areas. This finding contradicts previous studies showing that access to trash receptacles reduces overall litter in recreation sites (Schultz et al., 2013).

To understand this result, we examined the role of CH cleaning activities during campsite turnovers. A Mann–Whitney *U* test indicated that CH in campgrounds without trash receptacles (*Mdn* = 9) collected significantly more litter than CH in campgrounds with trash receptacles (*Mdn* = 5; *U* = 5653.5, *p* = < 0.001). This difference suggests that CH compensate for the lack of trash receptacles by removing more residual litter than their counterparts in areas with trash receptacles.

We further investigated CH impact on litter in the environment by comparing the unmanaged litter between camping areas (which had CH cleaning) and day use areas (which had no CH or staff cleaning). Day use areas have significantly more unmanaged litter than camping areas by mass density and accumulation rate (Table 6), and litter density by number is nearly significant as well. While day use areas may have more visitors than individual camp sites (we do not have those data), it is clear that having staff (volunteer or paid) whose job it is to collect litter significantly reduces the mass of unmanaged litter in the recreation areas.

Litter cleanup poses a financial burden for management organizations, and the economic contribution of volunteer CH litter-removal services can be considerable. Our estimates indicate that volunteer CH potentially saved Idaho State Parks more than $171,600 in litter-cleanup costs during the 2021 camping season alone. This represents significant monetary savings for a state park system operating under financial constraints. These estimates consider only litter-removal services and do not include the economic benefits of other CH activities such as visitor assistance, facility maintenance, and security presence.

Our statewide estimates suggest that CH programs have substantial cumulative impacts on litter management across Idaho’s public lands. While we estimated that CH remove > 111,000 pieces of litter annually from State Parks alone, the total impact across all public lands, including Bureau of Land Management and US Forest Service sites, is likely considerably higher. These estimates underscore the importance of volunteer CH programs for maintaining recreation area quality at scale. Policy decisions that reduce CH and other volunteer involvement may have unintended consequences for public land quality, as demonstrated by recent experiences in the Okanogan–Wenatchee National Forest where such cuts led to increased litter accumulation and reduced visitor enjoyment (Scruggs, 2025). Given both the economic savings and the substantial litter removal services these volunteers provide, investments in CH recruitment, training, and support represent a cost-effective approach to recreation area management.

## Conclusions

This study provides the first comprehensive assessment of litter characteristics, distribution, and management in Idaho’s recreational campgrounds, revealing important insights for land managers and policymakers. Our findings demonstrate that plastic litter dominates recreational areas by count (78.6%), significantly exceeding proportions found in roadways, waterways, and traditional waste streams. Litter types related to consumptive behaviors, such as plastic food wrappers, cigarette butts, various plastic containers are common across both state parks and national forests, suggesting that targeted education and mitigation efforts focused on food and beverage consumption waste would have broad applicability across public land types.

Despite experiencing population densities comparable to major urban areas during peak season (median 4,252 people/km^2^), Idaho’s recreational campgrounds maintain relatively low litter accumulation rates (2.13–2.18 kg/km^2^/day) compared to urban settings. This success appears to be related to intensive management of these areas, in which the role of the CHs is critical. Our comparison between camping areas (with CH services) and day use areas (without dedicated cleaning staff) demonstrates that CH presence substantially decreases litter density and accumulation rates, highlighting the effectiveness of volunteer-based management strategies.

The economic value of CH services is substantial. Conservative estimates indicate that volunteer CHs remove more than 111,150 pieces of litter (>277 kg) annually from Idaho State Parks alone, representing cost savings exceeding $171,600 per season when compared to paid-labor alternatives. These figures consider only litter removal and do not account for other valuable CH contributions such as visitor assistance and facility maintenance.

Our findings reveal no significant difference in litter levels between management approaches with or without trash receptacles, contradicting some previous research. However, this appears to be explained by compensatory cleaning efforts with CHs collecting significantly more litter in areas without trash receptacles. This suggests that active management through CH programs can be as effective as infrastructure-based solutions for litter control.

With 78% of litter being plastic by count these camping areas serve as potential hotspots for plastic flux into the environment. Therefore, maintaining and supporting CH programs should be considered an essential component of environmental stewardship in public lands. Policy decisions that reduce volunteer involvement may have serious unintended consequences for recreational area quality and environmental protection. As recreational use of public lands continues to increase, investing in and expanding CH programs represents a cost-effective strategy for maintaining environmental quality while providing significant economic benefits to resource-constrained management agencies.

Future research should employ standardized methodologies that account for urbanization levels and cleaning frequencies to enable better comparison of litter accumulation rates across different contexts. Additionally, investigating visitor education interventions targeted at the most common litter types identified in this study could provide valuable insights for reducing litter inputs at the source.

## Supplementary Information

Below is the link to the electronic supplementary material.

ESM 1 (DOCX 23.7 KB)

ESM 2 (XLSX 94.4 KB)

## Data Availability

All data generated or analyzed during this study are included in this published article and its supplementary information files.
