# Supplementary material for: Characterization and intervention impacts on litter across public land types in Northern Idaho
Source: Environ Monit Assess. 2026 Apr 28;198(5):520. doi: 10.1007/s10661-026-15342-w (PMC13124929; doi:10.1007/s10661-026-15342-w)
Supplement: Supplementary file 1 — (DOCX 23.7 KB) [file 10661_2026_15342_MOESM1_ESM.docx]

Supplemental Table 1: Estimated total unmanaged litter. This was determined as the multiplication of total campsites by average campsite areas, median litter accumulation rate, and average campground occupancy.

| Recreation Area | Campground | Total campsites | Average Campsite Area [km^2^] | Median Litter Accumulation  Rate  [#/km^2^/ days] | Average campground occupancy [days] | Estimated uncontrolled Litter  [#] |
| --- | --- | --- | --- | --- | --- | --- |
| Farragut | Waldron | 73 | .00034 | 3014 | 80 | 5949 |
|  | Whitetail | 61 | .00019 | 3644 | 81 | 3475 |
|  | Snowberry | 44 | .00019 | 2459 | 91 | 1841 |
| Heyburn | Hawley's landing | 52 | .00015 | 3293 | 64 | 1622 |
|  | Benewah | 39 | .00014 | 4772 | 56 | 1459 |
|  | Chatcolet | 37 | .00028 | 1922 | 42 | 824 |
| Ponderosa | RV | 50 | .00020 | 2726 | 98 | 2698 |
|  | Peninsula | 113 | .00018 | 1644 | 93 | 3041 |
| Lake Cascade | Poison Creek | 22 | .00026 | 2050 | 92 | 1095 |
|  | Van Wyck | 25 | .00029 | 5468 | 59 | 2347 |
|  | Ridgeview | 24 | .00027 | 2903 | 97 | 1804 |
| Payette NF | Last Chance | 23 | .00024 | 4201 | 18 | 414 |
|  | Upper Payette | 20 | .00016 | 3066 | 72 | 689 |
|  | Cold Springs | 30 | .00018 | 3777 | 25 | 499 |
| **Total** |  |  |  |  |  | **27,757** |

Supplemental Table 2: Estimated total CH litter collection during 100-day camping season based on campground campsite numbers, average litter collection rates, and estimated occupancy rates.

| **Recreation Area** | **Campground** | **CH avg daily litter rate [#/day]** | **Camping Season Days** | **Total campsites** | **Estimated Total Litter [#]** |
| --- | --- | --- | --- | --- | --- |
| **Farragut**** | Waldron | 0.96 | 100 | 73 | 6978 |
|  | Whitetail | 0.31 | 100 | 61 | 1865 |
|  | Snowberry | 0.47 | 100 | 44 | 2048 |
| **Heyburn** | Hawley's landing | 0.28 | 100 | 52 | 1435 |
|  | Benewah | 0.85 | 100 | 39 | 3303 |
|  | Chatcolet | 0.32 | 100 | 37 | 1193 |
| **Ponderosa**** | RV | 0.19 | 100 | 50 | 941 |
|  | Peninsula*** | N/A | 100 | 113 | N/A |
| **Lake Cascade**** | Poison Creek | 0.43 | 100 | 22 | 937 |
|  | Van Wyck | 1.06 | 100 | 25 | 2640 |
|  | Ridgeview | 0.38 | 100 | 24 | 911 |
| **Payette NF**** | Last Chance | 1.37 | 100 | 23 | 3156 |
|  | Upper Payette | 0.19 | 100 | 20 | 388 |
|  | Cold Springs*** | N/A | 100 | 30 | N/A |
| **Total**/***Avg*** |  | ***0.57*** | ***100*** | **613** | **25,796** |
| **Camping season in Idaho usually falls between Memorial Day and Labor Day (100 days). As opening day varies based on individual campground situation and weather, we used this as a standard to measure the average number of days any campground regardless of its location might be open.* | | | | | |
| ***This location has more campgrounds than the ones included in the study and the estimates do not represent the entire SP/NF but only the campgrounds included in this research.* | | | | | |
| ****No camp host data* | | | | | |
